# Can the introduction of a 12-lead ECG help reduce mortality in those presenting with foot ulceration to multidisciplinary diabetic foot clinics? An observational evaluation of a real-world implementation pilot in England

**DOI:** 10.1007/s00125-024-06134-3

**Published:** 2024-04-08

**Authors:** Jonathan Valabhji, Naomi Holman, Nicholas Collins, Robert J. Young, Paul Chadwick, Adam Robinson, Rahul Nayar, Satyan Rajbhandari, David V. Coppini, Marie-France Kong, Simon Ashwell, Ananth Nayak, Sanjeev Mehta, Chris Manu, Michael Edmonds, Catherine Gooday, Ketan Dhatariya

**Affiliations:** 1 NHS England, Wellington House, London, UK; 2 Department of Diabetes and Endocrinology, St Mary’s Hospital, Imperial College Healthcare NHS Trust, London, UK; 3 Department of Metabolism, Digestion and Reproduction, Imperial College London, London, UK; 4 Department of Epidemiology and Biostatistics, Imperial College London, London, UK; 5 School of Population Health, Royal College of Surgeons in Ireland, Dublin, Ireland; 6 Salford Royal NHS Foundation Trust, Salford, UK; 7 Birmingham City University, Birmingham, UK; 8 South Tyneside and Sunderland NHS Foundation Trust, Sunderland, UK; 9 Lancashire Teaching Hospitals NHS Foundation Trust, Preston, UK; 10 University Hospitals Dorset NHS Foundation Trust, Poole, UK; 11 University Hospitals of Leicester NHS Trust, Leicester, UK; 12 The James Cook University Hospital, South Tees Hospitals NHS Foundation Trust, Middlesbrough, UK; 13 University Hospitals of North Midlands NHS Trust, Stoke-on-Trent, UK; 14 Ealing Hospital, London North West University Healthcare NHS Trust, London, UK; 15 Diabetic Foot Clinic, Kings College Hospital NHS Foundation Trust, London, UK; 16 Norfolk and Norwich University Hospitals NHS Foundation Trust, Norwich, UK; 17 Norwich Medical School, University of East Anglia, Norwich, UK

**Keywords:** 12-lead ECG, ECG, Foot ulceration, Implementation, Mortality, Multidisciplinary diabetic foot clinics, QTc prolongation, Real-world

## Abstract

**Aims/hypothesis:**

The risk of dying within 2 years of presentation with diabetic foot ulceration is over six times the risk of amputation, with CVD the major contributor. Using an observational evaluation of a real-world implementation pilot, we aimed to assess whether for those presenting with diabetic foot ulceration in England, introducing a 12-lead ECG into routine care followed by appropriate clinical action was associated with reduced mortality.

**Methods:**

Between July 2014 and December 2017, ten multidisciplinary diabetic foot services in England participated in a pilot project introducing 12-lead ECGs for new attendees with foot ulceration. Inception coincided with launch of the National Diabetes Footcare Audit (NDFA), whereby all diabetic footcare services in England were invited to enter data on new attendees with foot ulceration. Poisson regression models assessed the mortality RR at 2 and 5 years following first assessment of those receiving care in a participating pilot unit vs those receiving care in any other unit in England, adjusting for age, sex, ethnicity, deprivation, type and duration of diabetes, ulcer severity, and morbidity in the year prior to first assessment.

**Results:**

Of the 3110 people recorded in the NDFA at a participating unit during the pilot, 33% (1015) were recorded as having received an ECG. A further 25,195 people recorded in the NDFA had attended another English footcare service. Unadjusted mortality in the pilot units was 16.3% (165) at 2 years and 37.4% (380) at 5 years for those who received an ECG, and 20.5% (430) and 45.2% (950), respectively, for those who did not receive an ECG. For people included in the NDFA at other units, unadjusted mortality was 20.1% (5075) and 42.6% (10,745), respectively. In the fully adjusted model, mortality was not significantly lower for those attending participating units at 2 (RR 0.93 [95% CI 0.85, 1.01]) or 5 years (RR 0.95 [95% CI 0.90, 1.01]). At participating units, mortality in those who received an ECG vs those who did not was lower at 5 years (RR 0.86 [95% CI 0.76, 0.97]), but not at 2 years (RR 0.87 [95% CI 0.72, 1.04]). Comparing just those that received an ECG with attendees at all other centres in England, mortality was lower at 5 years (RR 0.87 [95% CI 0.78, 0.96]), but not at 2 years (RR 0.86 [95% CI 0.74, 1.01]).

**Conclusions/interpretation:**

The evaluation confirms the high mortality seen in those presenting with diabetic foot ulceration. Overall mortality at the participating units was not significantly reduced at 2 or 5 years, with confidence intervals just crossing parity. Implementation of the 12-lead ECG into the routine care pathway proved challenging for clinical teams—overall a third of attendees had one, although some units delivered the intervention to over 60% of attendees—and the evaluation was therefore underpowered. Nonetheless, the signals of potential mortality benefit among those who had an ECG suggest that units in a position to operationalise implementation may wish to consider this.

**Data availability:**

Data from the National Diabetes Audit can be requested through the National Health Service Digital Data Access Request Service process at: https://digital.nhs.uk/services/data-access-request-service-dars/dars-products-and-services/data-set-catalogue/national-diabetes-audit-nda

**Graphical Abstract:**

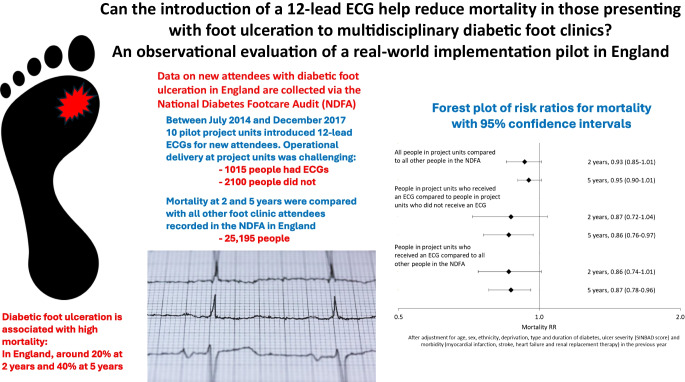

**Supplementary Information:**

The online version contains peer-reviewed and unedited supplementary material available at 10.1007/s00125-024-06134-3.


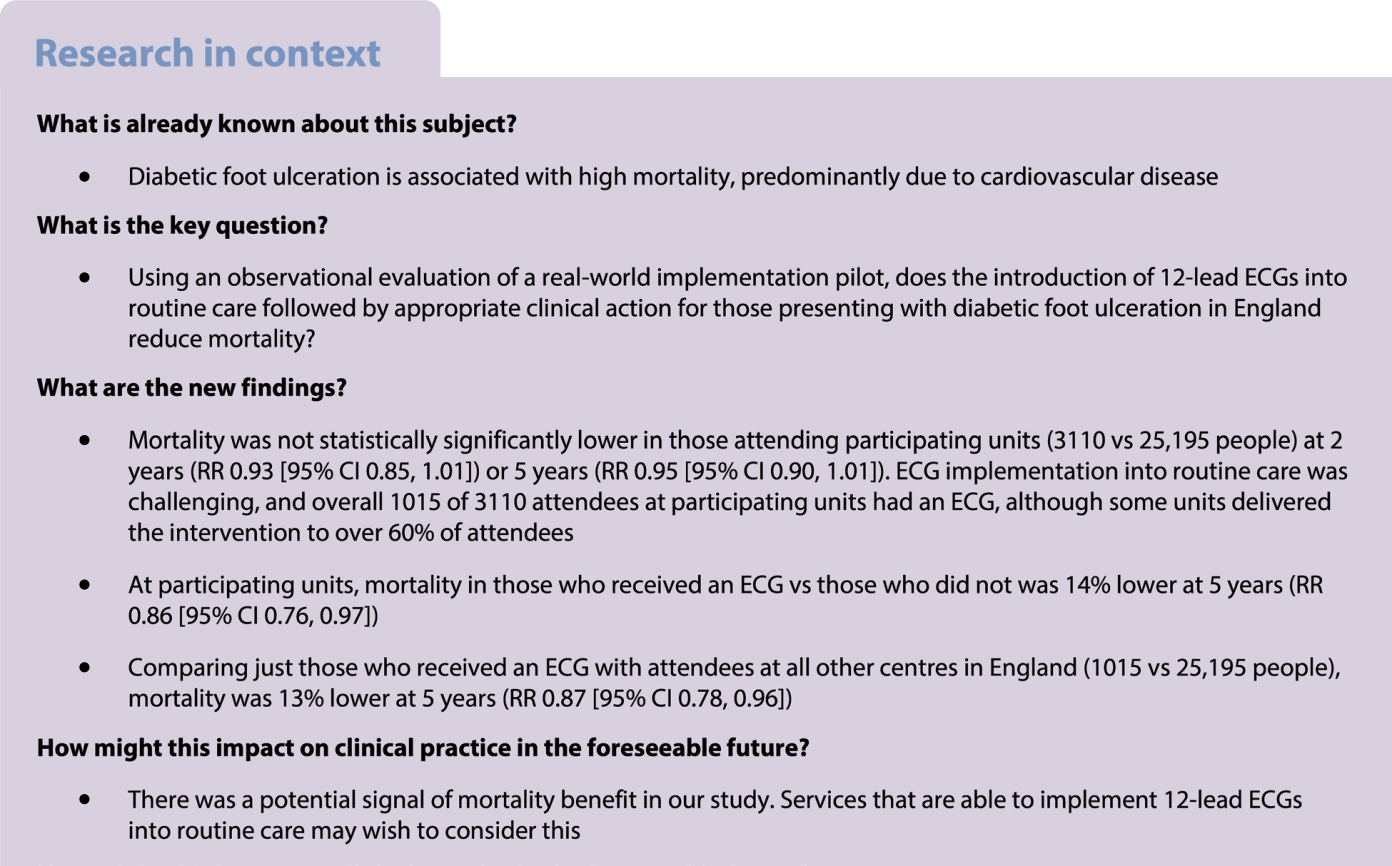


## Introduction

Limb loss is the most feared complication of diabetes, and foot ulceration its most common antecedent. Quality of diabetic foot disease care has therefore often been assessed by comparing amputation rates between services and between countries. Rates of major amputation within England have significantly decreased over the last decade and compare favourably to other countries [1]. However, the risk of dying within 2 years of presentation with diabetic foot ulceration is over six times the risk of amputation, making amputation-free survival a more appropriate clinical outcome measure [2]. As such, optimising both ulcer healing rates and patient survival rates are appropriate goals for care in multidisciplinary diabetic footcare services. CVD is a major determinant of reduced life expectancy in this patient group. Observational studies have previously suggested that QTc prolongation identified on 12-lead ECG in those presenting with diabetic foot ulceration is a potent independent predictor of both all-cause [3] and cardiac mortality [4], although the impacts of specific interventions to address this particular ECG abnormality have not been assessed.

Data accrued through routine care and routine clinical practice are now widely used for epidemiological analysis, including data collected as part of the National Diabetes Audit (NDA) in England [5, 6]. There is also increasing recognition that real-world observational data can contribute to the evidence around interventional effectiveness [7].

We aimed to assess whether the introduction of a 12-lead ECG into routine care for those with diabetic foot ulceration presenting to multidisciplinary diabetic footcare services in England followed by appropriate clinical action to address abnormalities in the 12-lead ECG was associated with reduced mortality.

## Methods

The National Diabetes Footcare Audit (NDFA) was established in England in July 2014 as an additional module of the NDA and has since continued with regular data collection. Each diabetic foot service in England is requested to enter data on new attendees presenting with diabetic foot ulceration. Data fields include date of first assessment, interval from presentation to any health professional with a new ulcer to first expert assessment (FEA), ulcer classification at FEA using the Site, Ischaemia, Neuropathy, Bacterial Infection, Area and Depth (SINBAD) score [8] and, at 12 weeks following FEA, whether the person is still alive, and if so, whether there is still active ulceration.

Using nationwide clinical networks to seek expressions of interest, 11 multidisciplinary diabetic foot services in England agreed to take part in a pilot project. The intention was to add a 12-lead ECG to the FEA. The ECGs were to be assessed for QTc prolongation or other ECG abnormalities, with appropriate clinical action taken where necessary. In order to minimise the burden of data collection, information on specific clinical actions taken was not recorded. All 11 services were required to collate the ECG data, in addition to populating the routine data fields required for the NDFA. Inception of the pilot project was synchronised with launch of the NDFA in July 2014, and data collection for the pilot project continued through to 31 December 2017. Regular tele-meetings between clinicians at the pilot sites helped to address any issues related to patient recruitment and data collection.

### Data sources

One of the 11 units did not proceed with participation in the ECG pilot project. The other ten units collected the following data on individuals who received a 12-lead ECG as part of their assessment: National Health Service (NHS) number (a unique patient identifier), date of the 12-lead ECG, the result of the 12-lead ECG (normal, prolonged QTc, other abnormality, or prolonged QTc plus other abnormality) and whether or not further action was taken as a result of the ECG findings (e.g. stopping medications that prolong QTc, referring to cardiology for rhythm disturbance or for evidence of active ischaemia). These data were linked at individual attendee level to the NDFA. The date of the ECG was not always recorded. Where the date of the ECG was recorded, data were linked if the ECG was performed within 7 days of the date of FEA recorded in the NDFA. If no date of ECG was recorded, linkage was limited to those individuals who only had one episode of care recorded in the NDFA between 14 July 2014 and 31 December 2017. An established linkage between NDFA and the core NDA [9] provided data from routinely collected electronic health records on age at FEA, sex (but not gender), ethnicity, type and duration of diagnosed diabetes, and socioeconomic status measured using quintiles of Index of Multiple Deprivation associated with the Lower Super Output Area derived from participant postcode [10]. NHS number was used to link to Hospital Episode Statistics, which provide data on all hospital admissions for people admitted to any NHS hospital in England [11]. Individuals who had been admitted to hospital for myocardial infarction (ICD-10 codes I21–22) (https://icd.who.int/browse10/2019/en), stroke (ICD-10 codes I61, I63–64, I679), heart failure (ICD-10 code I50) or renal replacement therapy (RRT) (ICD-10 codes N185, Z49, Z992; OPCS-4 codes M01, X40) (OPCS-4 CODE [datadictionary.nhs.uk]) in the year prior to inclusion in the NDFA were identified. NHS number was used to link to death registrations in England collated by the Office for National Statistics [12].

### Outcomes

The primary outcome was all-cause mortality within 2 years of FEA. The secondary outcome was all-cause mortality within 5 years of FEA. Applying data from a previous study assessing associations between QTc prolongation and mortality in those with type 2 diabetes presenting with foot ulceration [3], it was estimated that if clinical action on the basis of the ECG findings reduced 2 year mortality in those with QTc prolongation from 31.5% to 26.9%, then 4115 participants would be required in each group for 80% power to detect a reduction in mortality at the 0.05 significance level.

### Cohorts

Not all of the ten units were active in performing 12-lead ECGs over the entire recruitment period of the pilot project. Some units joined the pilot after July 2014, while others stopped recruiting prior to December 2017. The primary project cohort included all attendees who received an initial assessment for a diabetic foot ulcer in one of the ten units during the period between the dates of the first and last recorded 12-lead ECG performed at that unit (see Table 1 for a list of units and dates of participation). The comparator cohort was all other people included in the NDFA attending any of the other services in England (excluding all ten services in the pilot project) with a date of first assessment between 14 July 2014 and 31 December 2017. By December 2017, 204 specialist footcare services in England were participating in the NDFA. Based on service estimates of annual caseload at that point, case ascertainment within the NDFA was approximately 20%. Although there were no data on the representativeness by patient characteristics, modelling showed that the only patient characteristics associated with risk of death were age and smoking status [13]. All people included in the study were followed up for 5 years.

**Table 1:** Number of people in participating units and first and last recorded FEA that included an ECG

Participating unit	Date of first assessment including a 12-lead ECG	Date of last assessment including a 12-lead ECG	Number of people who had a 12-lead ECG matched to NDFA	Number of people in NDFA who did not have 12-lead ECG	% people receiving an ECG in pilot period
City Hospitals Sunderland Diabetes Foot Clinic	21/07/2014	11/09/2017	155	50	73.2
Ealing Hospital, North-West London University Healthcare NHS Trust	13/06/2017	22/12/2017	20	15	57.1
Imperial College Healthcare NHS Trust	15/10/2014	06/10/2017	95	40	70.4
King's College Hospital Diabetic Foot Clinic	15/07/2014	24/11/2017	200	480	29.4
Norfolk and Norwich University Hospital	15/08/2014	22/12/2017	225	535	29.6
Poole Hospital NHS Foundation Trust	25/09/2014	05/10/2017	80	105	43.2
Salford Royal NHS Foundation Trust	28/11/2014	19/12/2017	35	650	5.1
The James Cook University Hospital Diabetes Care Centre	14/07/2014	08/12/2017	170	90	65.4
University Hospitals of Leicester	06/07/2015	08/11/2017	35	135	20.6

Dates presented as day/month/year

During the pilot project, it transpired that not all new attendees presenting with diabetic foot ulceration at one of the participating units had a 12-lead ECG performed. Secondary intervention and comparator cohorts were therefore also defined. The 2 and 5 year mortality rates for people who received a 12-lead ECG within one of the participating units were compared with:

1. the 2 and 5 year mortality rates for people who received an FEA at the same units between the date of the first and last recorded 12-lead ECG performed at that unit, but who had not themselves received a 12-lead ECG
2. the 2 and 5 year mortality rates for all other people included in the NDFA from any of the other services in England (other than the ten units in the pilot project) with a date of first assessment between 14 July 2014 and 31 December 2017.

### Statistical methods

Statistical significance of the differences between categorical variables were tested using χ^2^ tests and the differences between medians were tested using Mann–Whitney *U* tests. Poisson regression models assessed the mortality RR at 2 and 5 years following FEA of those receiving care in a participating pilot unit at a time when the service was undertaking 12-lead ECGs as part of their assessment processes vs those receiving care in any other unit in England, after adjusting for age, sex, ethnicity, deprivation, type and duration of diabetes, ulcer severity (SINBAD score), and morbidity in the year prior to first assessment (hospital admission for myocardial infarction, stroke, heart failure and RRT). Two additional Poisson regression models were created. One compared the effect of having or not having a 12-lead ECG within the same unit after adjusting for the same variables on 2 and 5 year mortality. The other compared all those receiving an ECG in the pilot cohort with all other people included in the NDFA after adjusting for the same variables on 2 and 5 year mortality. Additionally, a Kaplan–Meier plot was derived to represent unadjusted mortality over the full 5 year follow-up period for those who underwent FEA in a pilot unit while they were undertaking 12-lead ECGs compared with those from other services in England.

### Information governance

The NDFA and NDA core data are collected and used in line with NHS England’s purposes as required under the statutory duties outlined in the National Health Service Act 2006 and Health and Social Care Act 2012. There is controlled access by appropriately approved individuals to data held on secure data environments entirely within the NHS England infrastructure. Data are processed for specific purposes only, including operational functions, service evaluations and service improvement. The data used to produce this analysis have been disseminated to NHS England under Directions issued under Section 254 of the Health and Social Care Act 2012. Ethics committee approval is not required for these specific purposes. Exclusion from the NDA is activated by an opt-out system, the National Data Opt-out service [14]. Approval to hold the NHS numbers of people receiving ECGs as part of the project, and to link these to the Office for National Statistics death registration data, was obtained from the NHS Health Research Authority Confidentiality Advisory Group [15]. All numbers taken from the NDFA and NDA are rounded to the nearest five to protect individuals’ confidentiality.

## Results

**Fig. 1:**
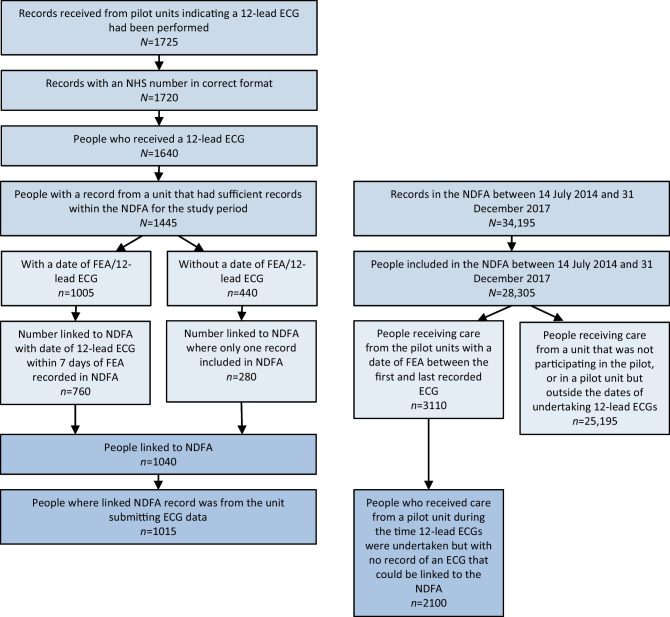
Flow chart of cohorts included in the analysis

Between July 2014 and December 2017, the ten units submitted data from 12-lead ECGs performed on 1725 diabetic foot ulcer FEAs; 1720 had the NHS number in the correct format to allow linkage to other datasets. Where the same individual had more than one episode of care, the first was used as the index episode: the 1720 episodes occurred in 1640 individuals. Linkage of the index episodes with NDFA recorded episodes was then undertaken. One unit provided details of 195 index episodes where an ECG had been performed but only three individuals could be linked to the NDFA indicating very low NDFA coverage, so all records from this unit were excluded, leaving 1445 index episodes. Of the 1445, 760 had a date when the ECG was performed and could be linked to an NDFA record which was within 7 days of the ECG, and 280 without a date of the ECG could be linked to an NDFA record where there was only one NDFA episode in the pilot study period. For a further 25 index episodes in which a 12-lead ECG had been performed, the corresponding NDFA episode was not at the same unit that had performed the ECG, and so these index episodes were also excluded. This left 1015 index episodes recorded in the NDFA in which a 12-lead ECG had been performed at one of the pilot units (Fig. 1 illustrates associated flow charts).

Between the date of the first and last recorded 12-lead ECG performed at each of the included units, a further 2100 individuals attended index episodes of care that were recorded in the NDFA during which there was no matched record of a 12-lead ECG having been performed.

Between July 2014 and December 2017, 25,195 individuals attended index episodes of care that were recorded in the NDFA at one of the other (non-pilot) footcare services in England, or at one of the pilot units outside the time in which 12-lead ECGs were taking place. For the purposes of these analyses, it was assumed that no 12-lead ECG was performed for these individuals.

The primary comparator cohort therefore included 3110 people recorded in the NDFA at one of the participating units, 1015 people who were recorded to have received a 12-lead ECG and 2100 people at the same units during the same time periods who had no recorded 12-lead ECG (numbers are rounded to the nearest 5); 25,195 people were recorded in the NDFA with an FEA between 14 July 2014 and 31 December 2017 at one of the other units in England (Table 2). Across all of the participating units, 32.6% of people presenting and recorded within the NDFA during the time that the service was undertaking ECGs received a 12-lead ECG but this varied from 5.1% to 73.2% across units (Table 1).

**Table 2:** Characteristics of the cohorts included in the analysis

Characteristic	In unit participating in pilot between project dates			Not in unit participating in pilot between project dates
	All	Received ECG	No known ECG	No known ECG
	*n*=3110	*n*=1015	*n*=2100	*n*=25,195
Sex				
Male	2110 (67.8)	705 (69.5)	1405 (66.9)	17,640 (70.0)
Female	945 (30.4)	290 (28.6)	655 (31.2)	7270 (28.9)
Unknown	55 (1.8)	15 (1.5)	40 (1.9)	285 (1.1)
Age (years)				
Median (IQR)	69 (58–78)	67 (57–77)	70 (59–79)	69 (58–78)
Type of diabetes				
Type 1	470 (15.1)	155 (15.3)	315 (15.0)	4165 (16.5)
Type 2 or other	2640 (84.9)	855 (84.2)	1785 (85.0)	21,030 (83.5)
Duration of diabetes (years)				
Median (IQR)	13 (8–20)	13 (8–19)	13 (8–20)	14 (8–20)
Ethnicity				
White	2525 (81.2)	810 (79.8)	1715 (81.7)	20,540 (81.5)
Asian	115 (3.7)	45 (4.4)	70 (3.3)	925 (3.7)
Black	140 (4.5)	50 (4.9)	90 (4.3)	580 (2.3)
Other	80 (2.6)	30 (3.0)	50 (2.4)	460 (1.8)
Missing	255 (8.2)	80 (7.9)	175 (8.3)	2685 (10.7)
Deprivation				
Most deprived	925 (29.7)	290 (28.6)	635 (30.2)	6340 (25.2)
2nd most deprived	640 (20.6)	220 (21.7)	420 (20.0)	5565 (22.1)
3rd most deprived	690 (22.2)	205 (20.2)	485 (23.1)	4985 (19.8)
2nd least deprived	505 (16.2)	175 (17.2)	330 (15.7)	4495 (17.8)
Least deprived	295 (9.5)	105 (10.3)	190 (9.0)	3475 (13.8)
Missing	55 (1.8)	15 (1.5)	40 (1.9)	335 (1.3)
SINBAD score				
Site	600 (19.3)	165 (16.3)	435 (20.7)	4340 (17.2)
Ischaemia	1095 (35.2)	360 (35.5)	735 (35.0)	8625 (34.2)
Neuropathy	2705 (87.0)	905 (89.2)	1800 (85.7)	20,050 (79.6)
Bacterial infection	1315 (42.3)	465 (45.8)	845 (40.2)	11,030 (43.8)
Area	1495 (48.1)	485 (47.8)	1010 (48.1)	12,560 (49.9)
Depth	610 (19.6)	205 (20.2)	405 (19.3)	4850 (19.2)
Score 3+	1445 (46.5)	495 (48.8)	960 (45.7)	11,520 (45.7)
Comorbidities				
Heart failure	300 (9.6)	75 (7.4)	225 (10.7)	2245 (8.9)
Myocardial infarction	70 (2.3)	20 (2.0)	50 (2.4)	495 (2.0)
Stroke	90 (2.9)	25 (2.5)	65 (3.1)	520 (2.1)
RRT	120 (3.9)	35 (3.4)	85 (4.0)	890 (3.5)
CKD stage 3+	785 (25.2)	255 (25.1)	530 (25.2)	6555 (26.0)
Died				
Within 2 years	600 (19.3)	165 (16.3)	430 (20.5)	5075 (20.1)
Within 5 years	1325 (42.6)	380 (37.4)	950 (45.2)	10,745 (42.6)

Data are *n* (%) or median (IQR)

CKD, chronic kidney disease

Table 2 demonstrates the characteristics of the cohorts included in the analyses. Median age was the same for those in the pilot units as for all other people included in the NDFA (69 years, IQR 58–78) but was lower in those who received a 12-lead ECG (67 years, IQR 57–77; *p*<0.005). There was a greater proportion of people living in areas in the most deprived quintile of socioeconomic deprivation in the pilot units compared with all other people included in the NDFA (*p*<0.001), but the distribution of deprivation was similar amongst those who did and did not receive an ECG as part of their FEA (*p*=0.291). The percentage of people with reported neuropathy was higher in the pilot units than amongst all other people in the NDFA (87.0% vs 79.6%; *p*<0.001). There were no significant differences in measures of ulcer severity between groups.

Of the 1015 people who received a 12-lead ECG, 505 (49.8%) had normal findings, 150 (14.8%) had prolonged QTc, 275 (27.1%) had other abnormalities and 65 (6.4%) had prolonged QTc and other abnormalities. Findings were not reported for 20 people (2.0%). It was reported that, in response to the ECG findings, clinical action was taken for 225 (22.2%) people; 15 (1.5%) people had data missing for this field.

Of all the people in the pilot units who received an FEA during the time periods in which ECGs were being performed, 600 (19.3%) died within 2 years and 1325 (42.6%) died within 5 years of the FEA (Table 2). Mortality in the pilot units was 16.3% (165) at 2 years and 37.4% (380) at 5 years for those who received an ECG and 20.5% (430) and 45.2% (950), respectively, for those who did not receive an ECG. In those who received a 12-lead ECG, mortality was lowest amongst those who had normal ECG findings (12.1% at 2 years and 29.6% at 5 years); intermediate in those with QTc prolongation and in those with other abnormalities (20.9% at 2 years and 48.0% at 5 years and 21.2% at 2 years and 43.6% at 5 years, respectively); and highest in those with both QTc prolongation and other abnormalities (25.4% at 2 years and 47.6% at 5 years).

Mortality for all other people included in the NDFA with FEA between 14 July 2014 and 31 December 2017 was 20.1% (5075) at 2 years and 42.6% (10,745) at 5 years. A Kaplan–Meier plot illustrates unadjusted mortality over the full 5 year follow-up period for those who underwent FEA in a pilot unit while they were undertaking 12-lead ECGs compared with those from other services in England (electronic supplementary material [ESM] Fig. 1).

**Fig. 2:**
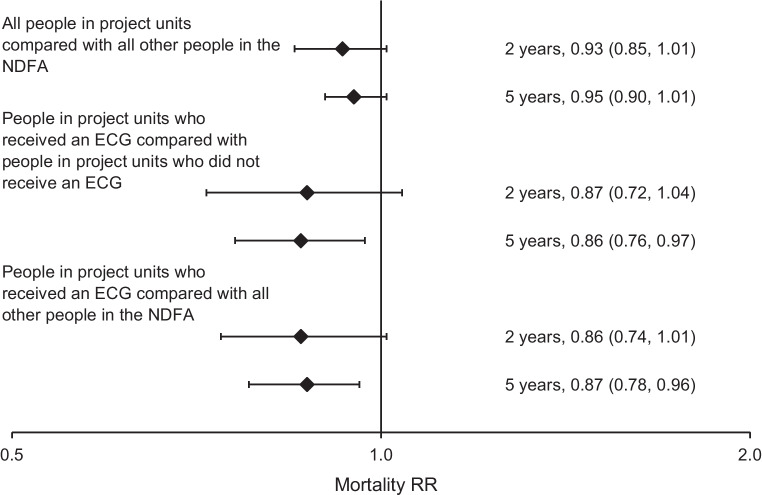
Forest plot of RRs for mortality, with 95% confidence intervals. Adjusted for age, sex, ethnicity, deprivation, type and duration of diabetes, ulcer severity (SINBAD score) and morbidity (myocardial infarction, stroke, heart failure and renal replacement therapy) in the previous year

In the primary analysis, after adjustment for age, sex, ethnicity, deprivation, type and duration of diabetes, ulcer severity (SINBAD score) and morbidity in the previous year, people who received an FEA in one of the pilot units during the time period that ECGs were performed had 7% lower mortality at 2 years (RR 0.93 [95% CI 0.85, 1.01]) and 5% lower mortality at 5 years (RR 0.95 [95% CI 0.90, 1.01]) compared with all other people included in the NDFA from any of the other units in England; neither of these differences reached statistical significance (Fig. 2).

In the secondary analyses, the people who received a 12-lead ECG within the pilot units were compared with:

1. People who received an FEA at the same units but did not receive a 12-lead ECG, between the date of the first and last recorded 12-lead ECG performed at that unit: there was no significant difference in 2 year mortality (RR 0.87 [95% CI 0.72, 1.04]) but 5 year mortality was significantly lower (RR 0.86 [ 95% CI 0.76, 0.97]) (Fig. 2).
2. All other people included in the NDFA from any of the other services in England with a date of FEA between 14 July 2014 and 31 December 2017: there was no significant difference in 2 year mortality (RR 0.86 [95% CI 0.74, 1.01]) but 5 year mortality was significantly lower (RR 0.87 [95% CI 0.78, 0.96]) (Fig. 2).

Mortality for those attending pilot units where over 60% of people included in the NDFA received a 12-lead ECG was not different from mortality for those attending pilot units where lower proportions received a 12-lead ECG, at either 2 years (RR 1.06 [95% CI 0.88, 1.27]) or 5 years (RR 0.98 [95% CI 0.86, 1.11]).

## Discussion

Inclusion of a 12-lead ECG into the routine care pathway for new attendees presenting to a specialist service with diabetic foot ulceration was not associated with a statistically significant overall reduction in 2 or 5 year mortality in attendees at the participating units. Despite intentions that all new attendees at the participating units would receive a 12-lead ECG, teams found it challenging to achieve in practice. Accordingly, across all the pilot units, an ECG was performed in one third of FEAs, although some units delivered the intervention to over 60% of attendees. There was a significantly lower 5 year mortality in those that had an ECG at the pilot units compared with those in the same units that did not. This may reflect selection bias within the clinic setting whereby, in a capacity-limited environment, people perceived clinically to have the most to gain were more likely to have a 12-lead ECG performed, or it may reflect a real benefit. Of note, those within the pilot units that had an ECG were significantly younger than those who did not. Such potential selection bias around who received a 12-lead ECG may persist even after adjusting for demographic characteristics, ulcer severity and comorbidities. This potential selection bias may also be relevant when interpreting the lower 5 year mortality in those that had an ECG at a pilot unit compared with those attending all other units in England.

### Strengths and limitations

The evaluation assessed the impact of a simple clinical intervention on individuals with diabetic foot ulceration presenting to diabetic footcare services in England, and the real-time challenges of implementation. However, low rates of implementation, with ECGs performed for 1015 of 3110 pilot unit attendees, meant that the evaluation was underpowered to detect differences in mortality at 2 years. The initial power calculation had determined that a minimum of 4115 individuals would be required to have the intervention. The overall 2 year mortality in England for attendees with foot ulceration turned out to be lower than that which had been applied in the power calculation (around 20% vs 31.5%), and the prevalence of QTc prolongation was lower than that seen in the Swedish cohort from which the power calculation was derived (21% vs 36%) [3], both factors further compromising power.

A longer duration of follow-up did demonstrate a statistically significant 13% reduction in 5 year mortality in those who had an ECG compared with those attending other units in England, but the potential for selection bias as to which attendees had an ECG in the pilot units makes it difficult to interpret this finding. Furthermore, the participating units were selected following expressions of interest to take part in the pilot project, without any form of randomisation, and it is possible therefore that these centres were particularly interested in diabetic foot disease and may have delivered overall better care. The fact that the percentage of people with reported neuropathy was higher in the pilot units may indicate more thorough clinical assessment and reflect overall better care, although this finding is difficult to reliably interpret. On the other hand, all of the participating units were hospital-based multidisciplinary footcare services where case mix may be more complex; community-based services also submit data to the NDFA, and may have less complex case mix, but were not represented in the participating units.

Unfortunately prescription data were not available in the NDA over the period of the pilot project, so it is not possible to ascertain whether prescription rates for cardiovascular risk modifying medications, such as statins, ACE inhibitors, beta blockers or aldosterone antagonists were different in the pilot units compared with other units in England.

Therefore, these results contribute to evidence that, in the very high-risk diabetic foot ulcer population, routine ECGs might be a route to improving the poor life expectancy, but they are far from definitive in this regard. An adequately powered RCT would be required. Nonetheless, an RCT will not necessarily address all of the implementation challenges that we have found when applying the intervention into live clinical environments. Although simple in concept, it involves additional equipment within the foot clinic setting, staff and patient time to perform the 12-lead ECG, a separate room in which to perform the ECG for the required privacy, and the physician time to both interpret the ECG and act on abnormal results. In some units, individuals were required to attend the cardiology unit for the 12-lead ECG to be performed, adding logistical challenges for those not ambulant, and possibly contributing to the selection bias as to who had an ECG. When carried out, an impressive 22% of the ECGs led to additional clinical actions. This may have been contributory to the lower 5 year mortality seen in these individuals compared both to individuals at the same units who did not have an ECG, and to individuals attending other units across England.

The analysis is limited to all-cause mortality and it is possible that the introduction of 12-lead ECGs to the FEA had a greater impact on some causes of death than others. Using routinely collated data to evaluate the impact of new clinical pathways or interventions has advantages of low cost. However, there are also challenges. Data available for analysis are limited to those recorded during the course of standard care and tend to have a higher proportion of missing data than those collected specifically for research purposes. In addition, when considering all ten units that performed 12-lead ECGs on new attendees, only 58% of all episodes of care could be included in the analyses, with a large proportion not linkable to NDFA episodes. The burden of data entry for the NDFA, as well as the logistics of performing, interpreting, recording and acting on the results of ECGs, proved difficult for teams to accommodate within the environments of busy multidisciplinary foot clinics.

### Conclusions

The evaluation confirms the high mortality seen in those presenting with diabetic foot ulceration, with around 20% mortality by 2 years and 40% by 5 years, comparable to other UK-based studies [16, 17]. Overall mortality at the participating units was not significantly reduced at 2 or 5 years, with confidence intervals just crossing parity. Implementation of the 12-lead ECG into routine care pathways proved challenging for clinical teams and few centres actively performed ECGs throughout the entire period of the pilot; the evaluation was therefore underpowered. The principle of piloting of service improvements, with evaluation using real-world data, is one that has been applied successfully and extensively in recent years by the NHS England Diabetes Programme to improve care delivery and outcomes nationally [18, 19]. However, the evaluated benefits of this current care pathway pilot and the challenges around its practical implementation suggest that it is not currently possible to make firm recommendations on its implementation nationally in England. Implementation of the intervention therefore requires further research and evaluation. Nevertheless, the signals of potential mortality benefit among those who had an ECG suggest that units in a position to operationalise implementation may wish to consider this.

### Supplementary Information

Below is the link to the electronic supplementary material.

Supplementary file1 (PDF 255 KB)

## Data Availability

Data from the National Diabetes Audit can be requested through the NHS Digital Data Access Request Service process at: https://digital.nhs.uk/services/data-access-request-service-dars/dars-products-and-services/data-set-catalogue/national-diabetes-audit-nda

## Abbreviations

FEA: First expert assessment
NDA: National Diabetes Audit
NDFA: National Diabetes Footcare Audit
NHS: National Health Service
RRT: Renal replacement therapy
SINBAD: Site, Ischaemia, Neuropathy, Bacterial infection, Area and Depth

## Authors’ relationships and activities

JV was the National Clinical Director for Diabetes and Obesity at NHS England from 2013 to September 2023. NH is funded by NHS England to use the National Diabetes Audit data to explore questions relevant to health policy. RY has been Clinical Lead for the National Diabetes Audit of England and Wales since 2003. DVC was clinical lead for diabetic foot services at Poole Hospital 2014–2017. CG was a member of the National Diabetes Footcare Audit Committee 2014–2021. All other authors declare that there are no relationships or activities that might bias, or be perceived to bias, their work.

## References

[1] Valabhji J (2020). Rapid access to multidisciplinary diabetes foot care teams. BMJ.

[2] Vadiveloo T, Jeffcoate W, Donnan PT (2018). Amputation-free survival in 17,353 people at high risk for foot ulceration in diabetes: a national observational study. Diabetologia.

[3] Fagher K, Löndahl M (2013). The impact of metabolic control and QTc prolongation on all-cause mortality in patients with type 2 diabetes and foot ulcers. Diabetologia.

[4] Wang S, He Y, Xu L (2018). Association between QTc interval prolongation and outcomes of diabetic foot ulcers: Data from a 4-year follow-up study in China. Diabetes Res Clin Pract.

[5] Barron E, Bakhai C, Kar P (2020). Associations of type 1 and type 2 diabetes with COVID-19-related mortality in England: a whole-population study. Lancet Diabetes Endocrinol.

[6] Holman N, Knighton P, Kar P (2020). Risk factors for COVID-19-related mortality in people with type 1 and type 2 diabetes in England: a population-based cohort study. Lancet Diabetes Endocrinol.

[7] Craig P, Campbell M, Bauman A (2022). Making better use of natural experimental evaluation in population health. BMJ.

[8] Ince P, Abbas ZG, Lutale JK (2008). Use of the SINBAD classification system and score in comparing outcome of foot ulcer management on three continents. Diabetes Care.

[9] Holman N, Knighton P, Wild SH (2021). Cohort profile: National Diabetes Audit for England and Wales. Diabetic Medicine.

[10] UK Government National Statistics (2019) English indices of deprivation 2019. Available from https://www.gov.uk/government/statistics/english-indices-of-deprivation-2019. Accessed 2 Sep 2023

[11] NHS Digital (2023) Hospital Episode Statistics (HES). Available from https://digital.nhs.uk/data-and-information/data-tools-and-services/data-services/hospital-episode-statistics. Accessed 2 Sep 2023

[12] Office for National Statistics (2023) Deaths. Available from https://www.ons.gov.uk/peoplepopulationandcommunity/birthsdeathsandmarriages/deaths. Accessed 2 Sep 2023

[13] NHS Digital (2019) National Diabetes Foot Care Audit, 2014-2018. Available from https://digital.nhs.uk/data-and-information/publications/statistical/national-diabetes-footcare-audit/2014-2018. Accessed 22 Sep 2023

[14] NHS Digital (2023) National Data Opt-Out. Available from https://digital.nhs.uk/services/national-data-opt-out. Accessed 22 Sep 2023

[15] NHS Health Research Authority (2023) Confidentiality Advisory Group. Available from https://www.hra.nhs.uk/about-us/committees-and-services/confidentiality-advisory-group/#:~:text=The%20Confidentiality%20Advisory%20Group%20%28CAG%29%20is%20an%20independent,Secretary%20of%20State%20for%20Health%20for%20non-research%20uses. Accessed 30 Sep 2023

[16] Walsh JW, Hoffstad OJ, Sullivan MO, Margolis DJ (2016). Association of diabetic foot ulcer and death in a population-based cohort from the United Kingdom. Diabetic Medicine.

[17] Saluja S, Anderson SG, Hambleton I (2020). Foot ulceration and its association with mortality in diabetes mellitus: a meta-analysis. Diabetic Medicine.

[18] Ross JAD, Barron E, McGough B (2022). Uptake and impact of the English National Health Service digital diabetes prevention programme: observational study. BMJ Open Diabetes Res Care.

[19] Barron E, Bradley D, Safazadeh S (2023). Effectiveness of digital and remote provision of the Healthier You: NHS Diabetes Prevention Programme during the COVID-19 pandemic. Diabetic Medicine.

