# Supplementary material for: Can the introduction of a 12-lead ECG help reduce mortality in those presenting with foot ulceration to multidisciplinary diabetic foot clinics? An observational evaluation of a real-world implementation pilot in England
Source: Diabetologia. 2024 Apr 8;67(7):1304–14. doi: 10.1007/s00125-024-06134-3 (PMC11153259; doi:10.1007/s00125-024-06134-3)
Supplement: Supplementary file 1 — Supplementary file1 (PDF 255 KB) [file 125_2024_6134_MOESM1_ESM.pdf]

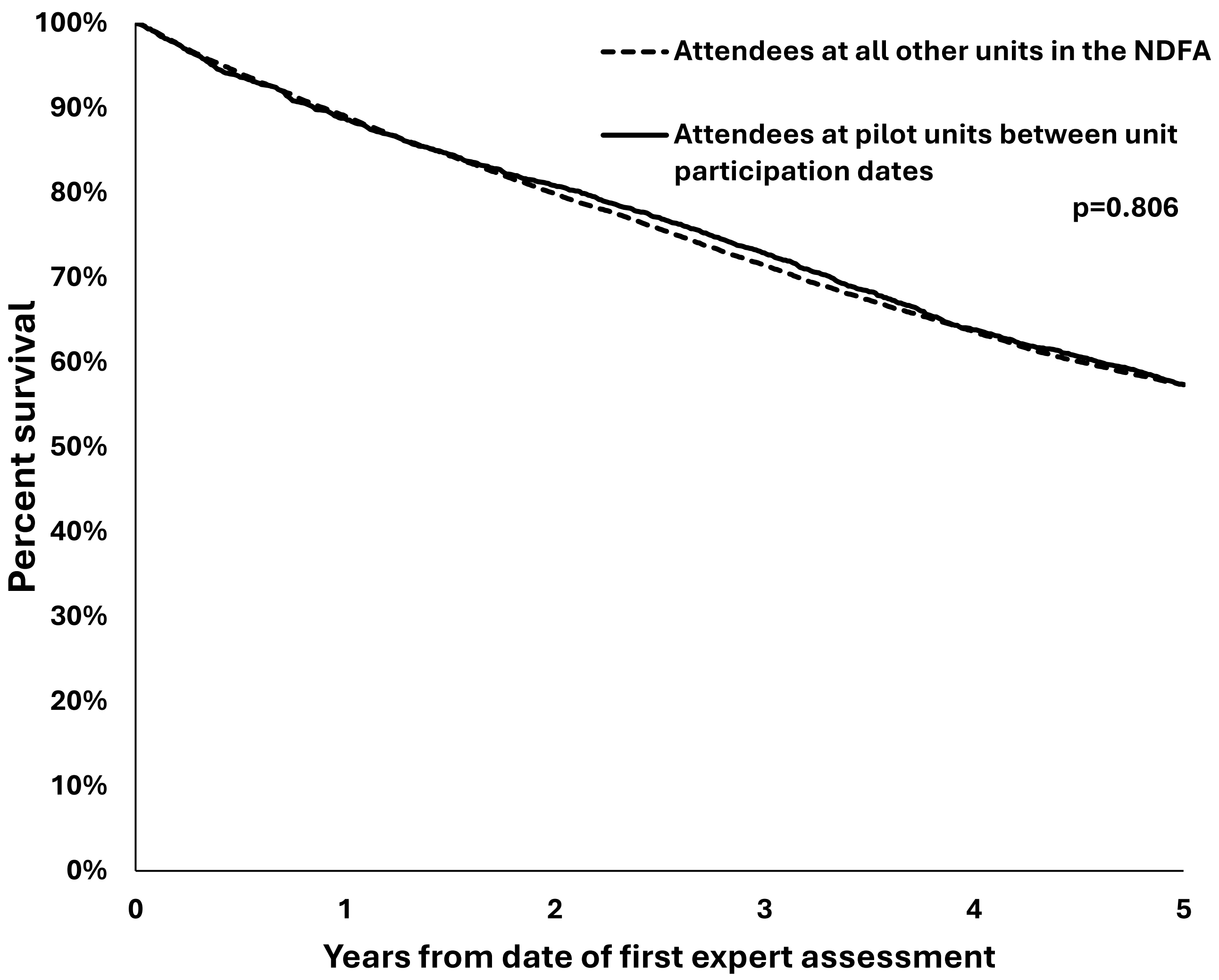

ESM Fig. 1: Kaplan–Meier plot representing unadjusted mortality over the full 5-year follow-up period for those who underwent first expert assessment in a pilot unit while it was undertaking 12-lead ECGs compared with those from other units in England participating in the National Diabetes Footcare Audit (NDFA)
